# Detecting and managing partial shorts in Cochlear implants: A validation of scalp surface potential testing

**DOI:** 10.1111/coa.13963

**Published:** 2022-08-01

**Authors:** Susan T. Eitutis, Yu Chuen Tam, Iwan Roberts, Chloe Swords, James R. Tysome, Neil P. Donnelly, Patrick R. Axon, Manohar L. Bance

**Affiliations:** ^1^ Cambridge Hearing Group, Department of Clinical Neurosciences University of Cambridge Cambridge UK; ^2^ Emmeline Centre Cambridge University Hospitals NHS Foundation Trust Cambridge UK; ^3^ Department of ENT Cambridge University Hospitals NHS Foundation Trust Cambridge UK

**Keywords:** Cochlear implant, electrical field imaging, electrode fault, partial short circuit, SCINSEVs, surface potential, ultra V1, V1 fault

## Abstract

**Objective:**

To investigate the value of scalp surface potentials to identify and manage partial short circuits to ground in cochlear implant electrodes.

**Design:**

A retrospective review of patients with suspected partial short circuits.

**Main outcome measure:**

Electrical output of individual electrodes was measured using scalp surface potentials for patients reporting a change in hearing function. Electrical output was compared to functional performance and impedance measurements to determine if devices with suspected partial short circuits were experiencing a decrease in performance as a result of reduced electrical output. Electrical output was checked in an artificial cochlea for two implants following explant surgery to confirm scalp surface potential results.

**Results:**

All patients with suspected partial short circuits (*n* = 49) had reduced electrical output, a drop in impedances to approximately ½ of previously stable measurements or to below 2 kΩ, an atypical electrical field measurement (EFI) and a decline in hearing function. Only devices with an atypical EFI showed reduced electrical output. Results of scalp based surface potentials could be replicated in an artificial cochlea following explantation of the device. All explant reports received to date (*n* = 42) have confirmed partial short circuits, with an additional four devices failing integrity tests.

**Conclusion:**

Surface potential measurements can detect partial shorts and had 100% correlation with atypical EFI measurements, which are characteristic of a partial short to ground in this device. Surface potentials can help determine the degree to which the electrode array is affected, particularly when behavioural testing is limited or not possible.

**Key Points**

1. In‐vitro testing confirmed that scalp based surface potential measurements could detect cochlear implant electrodes with reduced output and the degree to which electrical output was reduced (% output reduction).
2. Patients showing a decline in listening performance in combination with a significant drop in impedances should have testing to investigate for *suspected partial short circuits* including electrical field imaging (EFI), functional testing, neural response tests (NRI), and optional surface potential measurements.
3. Drops in functional performance in addition to an atypical EFI, drop in impedance and reduced electrical output are an indicator of partial short circuits within the electrode array.
4. Optional management for patients showing partial short circuits includes increasing threshold and comfort levels on affected electrodes, using the span function in the programming software to bypass faulty electrodes, deactivating affected electrodes and re‐implantation.
5. Scalp based surface potentials are recommended for patients who are unable to provide accurate feedback or who are unable to undergo device re‐implantation.

## 1 INTRODUCTION

Cochlear implants (CIs) are well accepted as an effective intervention for adult and paediatric patients with severe‐profound hearing loss. However, CIs are not infallible, and device functionality must be monitored over time to ensure they maintain optimal function. A replacement CI may be offered when devices no longer provide adequate auditory benefit, which cannot be resolved with routine adjustments or medical attention.

Re‐implantation is required in approximately 6% (range, 0.47–6.87%) of CI recipients.^1^ Standardised reliability reporting is used to classify any re‐implantation as a medical failure, device failure or inconclusive failure.^2^ Device failures account for the majority of re‐implantation cases, with 3.6% (range, 0.8–5.8%)^1^ of re‐implants being due to failure of components related to device function.^3^ Although device failure is rare, routine monitoring of the implant can ensure changes to performance are appropriately investigated and managed.

The most basic clinical test for monitoring device function is contact impedance measurement (telemetry). Impedances reflect the amount of ‘resistance’ to current flow in the cochlear environment. They are a measure of the voltage generated on a given electrode relative to the return ground for a known current injection, and represent a snapshot of the electrode‐biological interface. Changes to impedances can reflect differences in the cochlear environment, such as the conductivity of surrounding perilymph, soft tissues and bone, and the amount of tissue or bone growth.^4^, ^5^, ^6^ Impedances are essential to tracking the integrity of the electrode array and leads, where open circuits (impedances >20 kΩ) or short circuits (impedances <1 kΩ) are potential indicators of device faults.^6^, ^7^ Electrodes identified as faulty through impedance monitoring can be managed by deactivating the affected electrodes. However, electrodes with partial short circuits, or exhibiting large changes in otherwise stable measurements, pose a more challenging problem.

The recent voluntary field corrective action for the Advanced Bionics HiRes Ultra Version 1 (V1) devices highlights the need to better understand low impedances and how to identify and manage patients already implanted with these devices.^8^ Patients with V1 devices are at risk of developing partial short circuits (also termed ‘Ultra V1 fault’), particularly on basal electrodes. These partial short circuits can lead to altered sound perception and functional changes. However, knowing which electrodes are affected, and to what degree, requires additional testing as partial short circuits are not flagged during routine impedance tests, are difficult to diagnose with clinical tests, and can only be confirmed by explanting the device.

Identifying electrode faults caused by changes to impedances and electrical output has previously been successful using surface potential measurements.^9^ Surface potentials measure electrical activity of the implant and are far larger than neural responses. More recently, Grasmeder applied a similar approach to detecting extra‐cochlear and migrating electrodes.^10^ Here we present our clinical experience managing suspected Ultra V1 device faults detected using surface potentials.

## 2 METHODS

### 2.1 Patients

Retrospective chart reviews for 70 Advanced Bionics Ultra V1 devices (*n* = 43 Mid‐Scala, 21 SlimJ, 6 3D‐SlimJ implants) were completed for patients attending appointments between January 2019 and May 2021. Thirteen of these devices served as controls, and were collected from routine visits where the patients had no reported hearing changes. The remaining patients reported a decline in hearing and attended appointments to check for indications of electrode failure. Repeat testing was completed for four implants as part of on‐going monitoring for Ultra V1 device faults.

This data analysis was registered as a retrospective audit of managing implants with suspected Ultra V1 failures (PRN: 10031) and did not require ethical approval in our institution. Implants were only *suspected* to have partial short circuits (Ultra V1 failure) because definitive confirmation of this failure mode required explanting the device.

### 2.2 Clinical tests

All 70 implants completed the same set of tests for hearing performance/functional testing (soundfield aided thresholds (SFAT), auditory speech sound evaluation (ASSE), and Bamford‐Kowal‐Bench sentences (BKB)) and device function (impedance measurements, electrical field imaging (EFI) with Volta (version 1.1.1) or Active Insertion Monitoring (AIM) system, and surface potential measurements). For those who could not complete functional testing, neural response imaging (NRI) was repeated. For a small number of patients, X‐rays were completed to rule out electrode migration.

### 2.3 Electrical field imaging (EFI)

EFIs provide a view of complex impedances by measuring voltage generated on adjacent non‐stimulating electrodes during impedance testing.^11^ They are a function of transverse and longitudinal impedances in the cochlea, and the impedance of the electrode. No current is flowing in the adjacent measuring electrodes, so they are not dominated by near‐field electrode‐electrolyte effects.^12^ EFIs are represented graphically (Volta), or as a heatmap (AIM and ‘*EFI Analysis Tool*’ v1.2 software), where the height of the line or colour of the heatmap reflects the voltage measured at that electrode. Normal EFI measurements show a gradual, uniform reduction in current voltage moving from apical to basal electrodes and a relatively even spacing of the coloured lines along the *y*‐axis. However, electrode 1 *may* show a non‐uniform reduction in current due to a software artefact on the Volta system, which is not consistent across Volta measurements, and not present with the AIM system. If only electrode 1 showed a non‐uniform reduction on Volta, the EFI was considered normal.

### 2.4 Surface potential measurements

**FIGURE 1:**
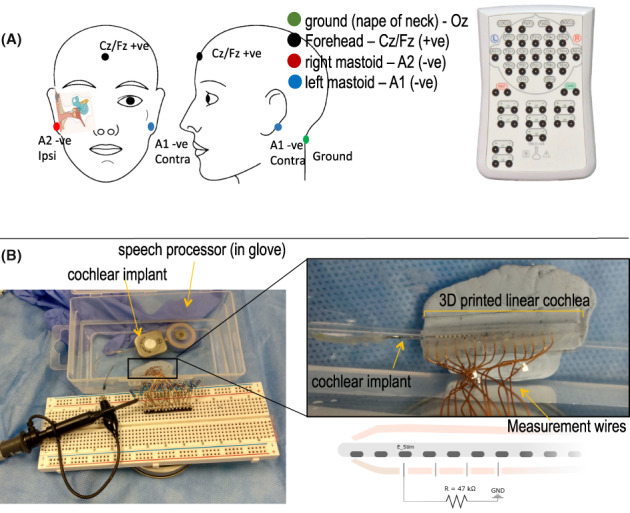
Measurement setup for determining electrode output. (A) Electrode montage for surface potentials on the scalp (Electrode montage → +ve forehead (CZ), −ve ipsi and contralateral mastoids (A1 and A2), and ground nape (Oz). (B) Experimental setup, and diagrammatic representation, for near‐field voltage measurements in the artificial cochlea. Here the stimulating electrode (*E*_stim_) is aligned to the measurement wire to perform the near‐field voltage measurement.

Surface potential measurements were completed to determine the output voltage (hereafter referred to as ‘output’ or ‘electrical output’) of each electrode using the Nicolet EDX 5ch system, with an electrode montage as shown in Figure 1A. Measurements were initially performed using a single‐channel approach on the ipsilateral mastoid until October 2020, after which a two‐channel approach was used due to a portion of paediatric patients showing reduced outputs on basal electrodes (Figure S1). Changing to a two‐channel approach limited the effect of possible electrical dipole shifts, caused by the geometry of the basal turn relative to the positional difference of the measurement electrodes, being misinterpreted as reduced electrical output. Using a two‐channel approach, the inverting signal from the ipsilateral and contralateral mastoids (A1 and A2) were measured simultaneously and compared against the non‐inverting forehead (Cz/Fz) electrode.

Electrode output was set using the Advanced Bionics SCLIN 2000 programming software and PSP speech processor plus headpiece. Output for all 16 electrodes was measured separately at 30, 60 and 120 current units (cu) using a 75 μs pulse. Traces typically stabilised after 10–20 recordings and had low noise floors. However, to maximise certainty a minimum of 150 traces were performed for each output level.

The output voltage (microVolts, μV), calculated as the maximum distance from baseline to peak, determined the positive and negative phase for each recording (16 electrodes, 3 output levels). Values were plotted using MS Excel® to provide a visual representation of output voltage across the electrode array. For clinical use, a graph with ‘normalised output’ was generated by averaging the measured voltage across 3–4 normal functioning electrodes (typically apical electrodes) to determine the percentage output reduction.

For statistical comparisons, ‘normalised output’ was based on the maximum measured output voltage across each electrode array ($\left(\frac{\text{electrode output}\;\left(\mathrm{\mu V}\right)}{\max\text{implant output}\;\left(\mathrm{\mu V}\right)}\right)*100=\text{normalised output}\;\left(\%\right)$). Where available, measurements from the contralateral mastoid only were used because they did not show the same voltage reduction on basal electrodes as ipsilateral measurements. Extra‐cochlear electrodes, or those showing a clear phase change, were removed from the analysis. Data was compared between (1) control implants, (2) implants with increasing impedances, (3) electrodes with ‘normal or borderline’ EFI responses on devices with atypical EFIs, and (4) electrodes with ‘atypical’ EFI responses on devices with atypical EFIs. Electrodes have ‘normal or borderline’ responses if they showed even spacing and a uniform reduction in current in the areas where the EFI had not collapsed. In contract, electrodes with ‘atypical’ responses clustered lower on the *y*‐axis and appeared as dark blocks on the EFI heatmap. The Kruskal–Wallis test, followed by the pairwise Wilcox test was used to compare output between these groups.

### 2.5 Near‐field voltage in artificial cochlea

Electrode output measurements were repeated in an artificial cochlea, using a fully functioning electrode array and two devices explanted due to suspected partial shorts. Results were compared to scalp surface potentials to ensure that clinical measurements were providing valid estimates of actual reduced electrical output, and not because of spatial vector shifts or other causes.

The near‐field voltage (μV) to each electrode contact was measured within a 3D printed linear cochlea model set‐up as developed by Jiang et al.^13^ The explanted CI was placed within a 3D printed linear cochlea with 14 copper wires connected in parallel to ground via 47 kΩ resistors (Figure 1B). Sequentially, each electrode was aligned against one measurement wire to record the near‐field peak‐to‐peak voltage when stimulated at 412 cu with 70 μs pulse width stimulus levels (3.5 times higher than the levels used for scalp measures to overcome the electrical noise from the artificial cochlea) using a Picoscope 2203 2‐channel oscilloscope controlled by Picoscope 6.14 software. Voltage measurements were taken in triplicate, averaged and normalised to maximal voltage measured along the array to compare relative voltage output and determine any faulty electrodes.

## 3 RESULTS

**FIGURE 2:**
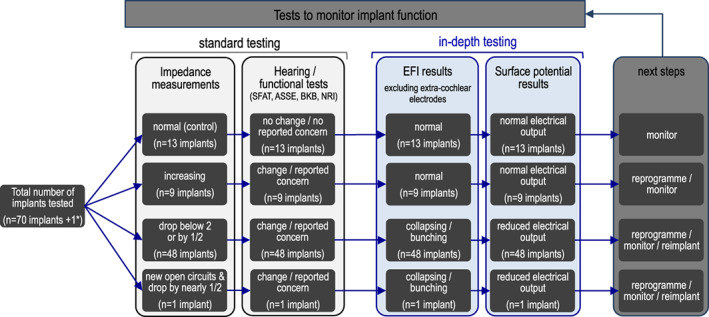
Monitoring tests and outcomes for patients with the Advanced Bionics HiRes Ultra V1 implant. A total of 70 implants had in‐depth and standard clinical tests completed to monitor for signs of partial short circuits. *1 implant initially presented with increasing impedances, had repeat surface potentials due to a drop in impedances 12 months after initial testing. ASSE, auditory speech sound evaluation; BKB, Bamford‐Kowal‐Bench sentences; NRI, neural responses; SFAT, sound field aided testing

Routine and in‐depth clinical tests were reviewed for 70 devices (Figure 2, Table S1). Hearing concerns were reported for 57 of these devices, while the remaining 13 implants served as controls. Partial short circuits were suspected for 49 devices due to dropping impedances. In‐depth testing revealed that only the 49 devices with suspected partial short circuits had atypical EFI and surface potential results.

### 3.1 Impedances

Patients who reported a change in function often showed either increasing or decreasing impedances. Patients with *increasing* impedances had a gradual or sudden increase to greater than 10 kΩ, typically in the basal region and described the sound as ‘blurred’ and ‘no longer clear’. Patients with *decreasing* impedances showed gradual or sudden drops to half of previously stable values or <2 kΩ and reported a ‘loss of clarity’ and drop in volume. Drops in impedance were most often on basal electrodes, however, were also been detected in apical only regions or across the whole array.

### 3.2 Electrical field imaging (EFI)

**FIGURE 3:**
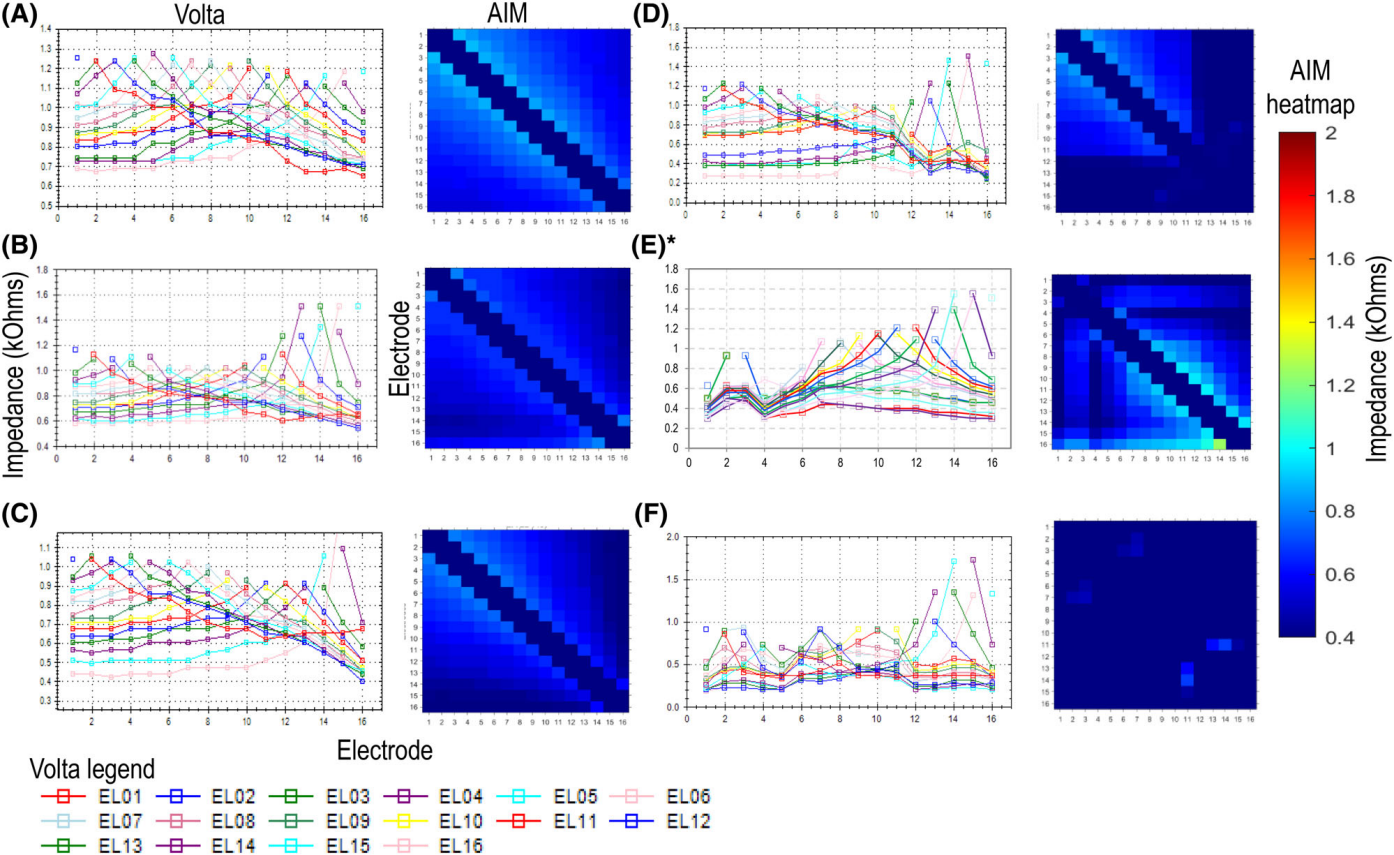
Representative examples of EFI measurements from Volta (coloured line graphs) and AIM system (heatmap). The following cases are presented: (A) normal, fully inserted electrode array. (B) Fully inserted electrode array with increasing impedances on basal electrodes. (C) Electrodes 14–16 extra‐cochlear. (D) Suspected partial shorts on basal electrodes. (E) Suspected partial shorts on apical electrodes. (F) Suspected partial shorts on apical, mid and basal electrodes. Due to a software artefact on the Volta system, electrode 1 only may show a non‐uniform reduction in impedances. *No EFI available on Volta for patients with apical faults, replotted from AIM data in format similar to Volta.

Representative examples of EFI traces and heatmaps can be seen in Figure 3. All 13 control implants had normal EFIs for intra‐cochlear electrodes (Figure 3A). Implants with increasing impedances (*n* = 9) also had normal EFIs, however with a stretched appearance (Volta) or brighter colour (AIM) on electrodes with high impedances (Figure 3B). Extra‐cochlear electrodes appeared as a change or drop in the slope in addition to higher contact impedances or as darker colours on the heatmap for contacts outside of the cochlea (Figure 3C).^11^

All patents with suspected partial short circuits exhibited EFI measurements that ‘collapsed’, indicated by drops in slope and uneven electrode spacing (Volta) or large dark sections or lines on the heatmap (AIM) (Figure 3D–F). Collapsed responses were most commonly isolated to basal electrodes (Figure 3D), however in some were also observed in apical regions (Figure 3E) or across the whole array (Figure 3F).

### 3.3 Surface potential measurements

**FIGURE 4:**
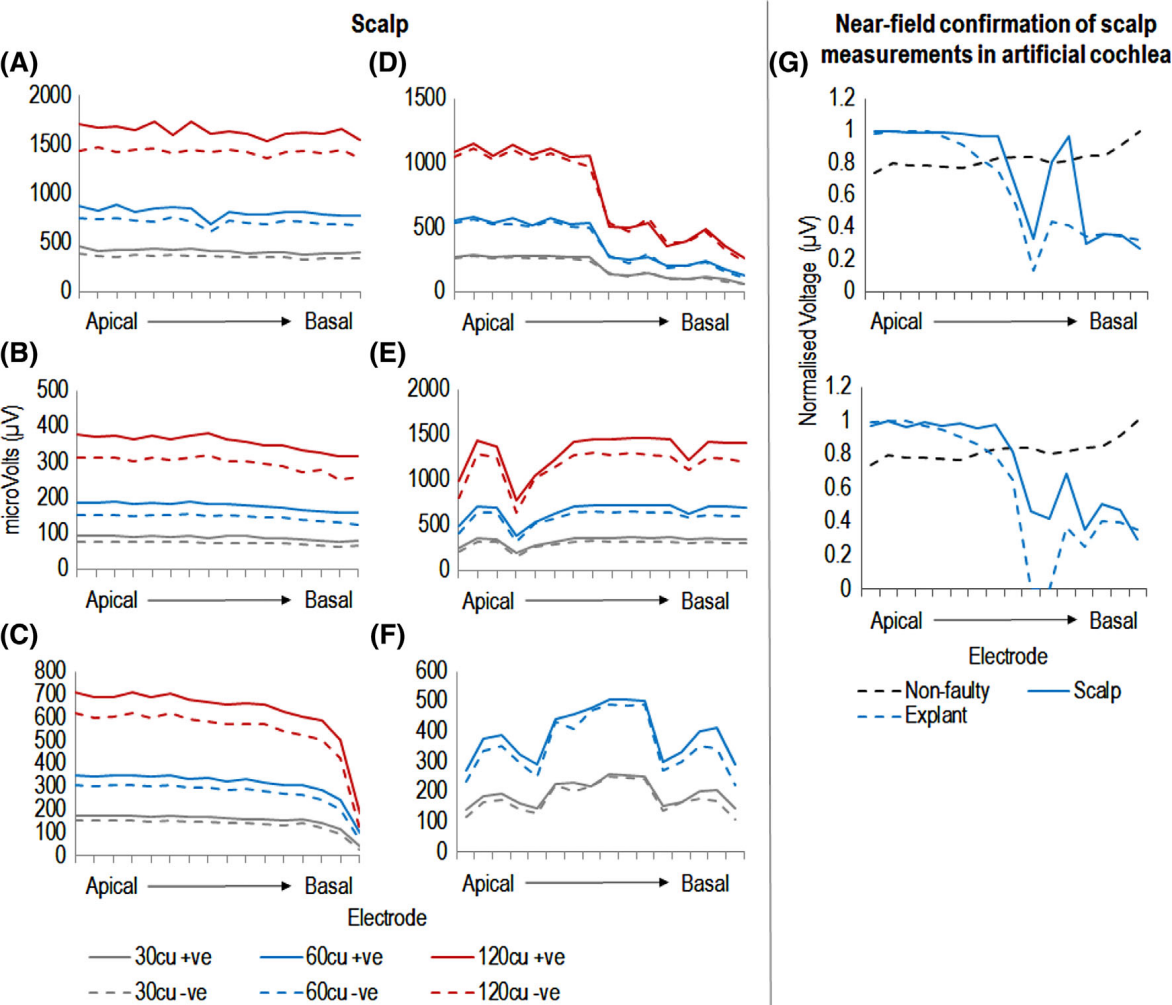
Representative examples of voltage output measurements taken on the scalp (A–F) and in an artificial cochlea following explant surgery (G). The voltage output (μV) for the positive phase (solid line) and negative phase (dotted line) for scalp‐based surface potentials are shown for three presentation levels. Please note the *y*‐axis scaling differs in each plot based the individual responses for each patient. The following cases are presented: (A) Typical, fully inserted electrode array. (B) Fully inserted electrode array with increasing impedances. (C) Electrodes 14–16 extra‐cochlear. (D) Suspected partial shorts on basal electrodes. (E) Suspected partial shorts on apical electrodes. (F) Suspected partial shorts on whole array. (G) Normalised peak near‐field voltages measured in an artificial cochlea (blue‐dashed) compared to scalp measurements taken prior to explantation (blue‐solid). Near‐field voltages of a control, non‐faulty implant are shown using a black dashed line.

Representative examples of electrical output across the array are shown in Figure 4. Devices were categorised as ‘normal output’ if all intra‐cochlear electrodes showed equal electrical output, or ‘reduced output’ if output was ≥20% less current on at least one intra‐cochlear electrode. All implants with stable or increasing impedances had normal surface potential output, and all implants with suspected partial short circuits had at least one electrode with reduced output. Repeat X‐rays for seven implants confirmed that reduced electrical output was not due to electrode migration/extra‐cochlear electrodes. Additionally, surgeons confirmed a full electrode insertion by visual inspection during re‐implantation surgery.

### 3.4 Comparing tests of device function

**FIGURE 5:**
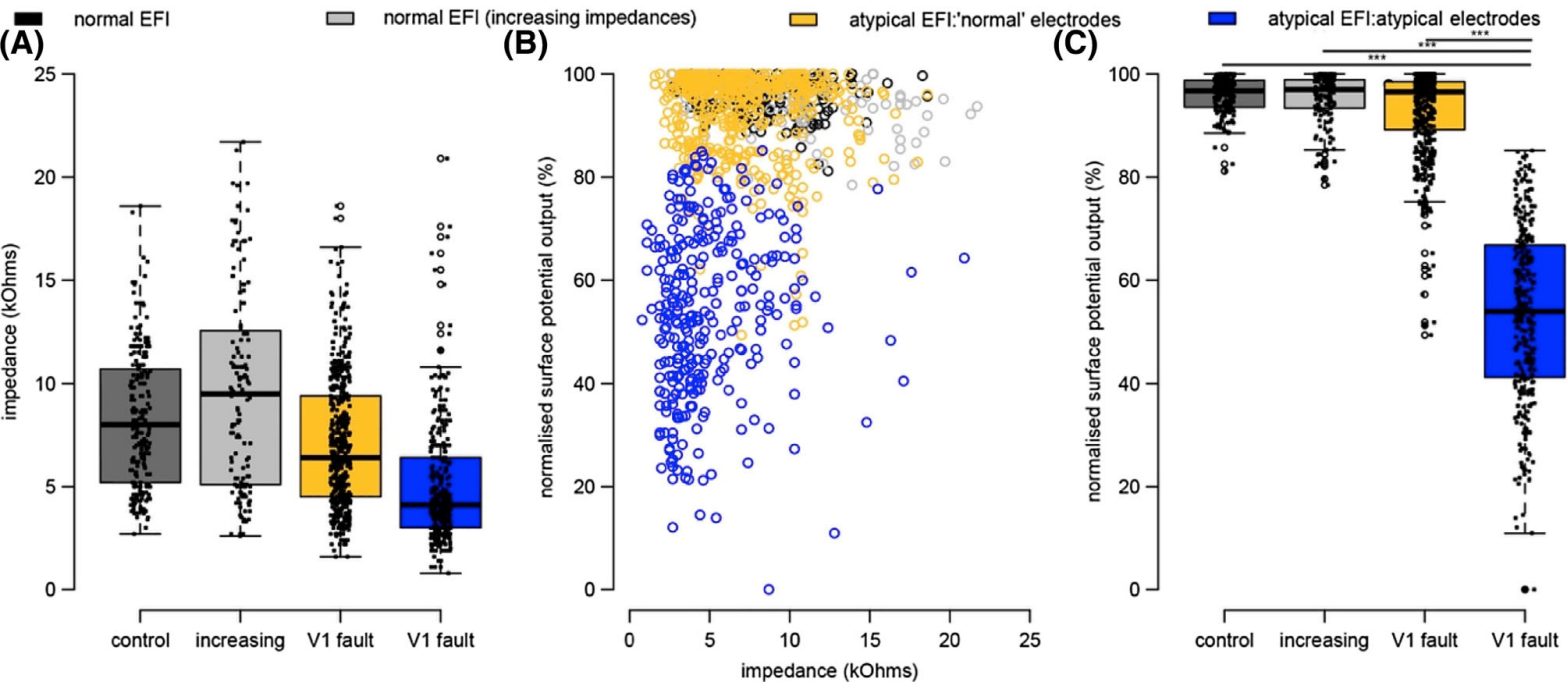
A comparison of impedances, EFIs and normalised surface potential tests for identifying electrodes with partial short circuits. Results have been grouped based on their EFI measurements, where control devices (dark grey) and those with increasing impedances (light grey) were observed to have normal EFIs, and devices with suspected partial short circuits (Ultra V1 faults) had atypical EFIs. For devices with suspected Ultra V1 faults, electrodes were categorised as showing ‘normal or borderline’ responses (gold) or clearly atypical response (blue) based on the EFI. (A) Impedance ranges based on EFI group. (B) Normalised surface potential output as a function of impedances. (C) Normalised surface potential output by EFI group. Individual electrodes with suspected partial short circuits had significantly reduced electrical output compared to all other groups. (***p < .001)

Impedances, EFIs and surface potentials were not equally consistent at identifying potentially problematic electrodes (Figure 5). A wide range of impedances was observed across all devices, including on electrodes with atypical behaviour on EFIs (Figure 5A). A wide range of electrical output was also observed across impedance values (Figure 5B).

In contrast, only electrodes with suspected partial short circuits had reduced electrical output (Figure 5C; Kruskal–Wallis, paired Wilcox test *p* < 2*e*−16) where 95.5% of atypical electrodes (*n* = 296 electrodes) had ≥20% reduction in electrical output. The remaining 4.5% (*n* = 14 electrodes) were reduced between 15 and 19%. Electrodes with apparent ‘normal or borderline’ behaviour, on the devices with atypical EFIs, had nearer normal output but more variation compared to devices with normal EFIs (Kruskal–Wallis, paired Wilcox test *p* < 0.025).

### 3.5 Validation of surface potentials

Electrical output was recorded in an artificial cochlea for explanted devices (Figure 4H). Measurements with one normally functioning implant showed similar output across all 16 electrodes. For two implants with partial short circuits, surface potentials with the artificial cochlea showed a similar although greater reduction in electrical output compared to scalp surface potentials. This is likely due to further progression of the fault in the 2‐months between clinical surface potentials and explant surgery.

### 3.6 Repeat tests

Four devices had all tests repeated 4–12 months after initial testing. One control device had stable results for functional tests, impedances, EFIs and surface potentials. Two devices suspected of having partial shorts showed further drops in impedances, more atypical EFIs, and further reduction of electrical output. Finally, one device previously with increasing impedances and normal EFI/surface potentials, presented with drops in functional tests, drops in impedances, atypical EFI and reduced electrical output.

### 3.7 Clinical management

Due to the significant impact on clinical function and atypical electrode behaviour, all 49 cases displaying the four key features of impedance drop, functional change, atypical EFI and reduced output were treated as ‘Ultra V1 failures’ and offered a replacement CI. To date, 44 of these implants have been replaced. Advanced Bionics has *confirmed* partial short circuits for 42 devices, with the remaining two explant reports yet to be returned. Additionally, four implants not yet explanted, have failed integrity testing showing patterns ‘consistent with the V1 failure mode’.^14^ We are therefore confident that the combination of functional testing, EFIs and surface potentials can robustly identify partial short circuits (Figure 2).

## 4 DISCUSSION

In our series, all implants showing (1) a drop in impedance by half or to <2 kΩ, (2) change in one or more functional measure (or NRI^15^), and (3) an atypical EFI were suspected as having a V1 failure. Scalp based surface potentials confirmed that only devices with atypical EFIs had reduced electrical output. Explant reports and integrity tests confirmed partial short circuits for 46 out of 49 suspected Ultra V1 failures. Confirmation of partial short circuits for the remaining three implants are pending, however these are likely to be Ultra V1 failures given the consistent clinical presentation across all 49 devices.

We have confirmed, both in the lab and using scalp based measurements, that surface potential testing effectively determines which electrodes are affected by partial short circuits, and to what degree electrode output is impacted. To our knowledge, this is the first time that reductions in scalp potential output in‐vivo have been confirmed in‐vitro as being due to reduced electrode voltage, with comparisons of magnitudes of drop (range, 20–100% reduced output). This further validates surface potential measurements as a diagnostic clinical tool. We suspect the further reduction in‐vitro versus in‐vivo was due to rapid progression of affected electrodes, which is common with this fault.

### 4.1 Management of patients with partial short circuits

The reduced electrical output caused by partial short circuits can significantly impact hearing performance. Management of partial short circuits will depend on the degree to which the electrodes are affected. For patients with a clear decline in performance, threshold measurements as well as loudness growth should be checked for electrodes of concern. All Ultra V1 devices should have regular monitoring due to the progressive nature of this failure mode, and management may include increasing the volume on affected electrodes, deactivating affected electrodes, or replacing the internal electrode array (Table 1).

**TABLE 1:** Management options for patients with partial short circuits who demonstrate a clear decline in performance

	Criteria
1.	Electrodes showing only slight differences in loudness growth should be balanced with the remaining electrodes and current levels elevated slightly if required.
2.	‘Spanning’, which enables current steering between non‐adjacent electrodes in order to bypass a faulty electrode contact, should NOT be used if neighbouring electrodes have altered loudness growth as sound quality may continue to be affected.
3.	Electrodes where sound can no longer be detected, or where loudness growth is considerably different from the rest of the electrode array should be deactivated (check using behavioural measures, or analyse amplitude growth of NRI if behavioural testing is not possible).
4.	If more than three electrodes require deactivation or performance continues to decline after programming, patients should be brought to team discussion for re‐implantation options.

Surface potentials are not necessary if impedances, EFI and functional changes are consistent with partial short circuits. However, they may be a useful adjunct if additional evidence of a device fault is required (for Ultra V1 or other failure modes) or if the patient is unable to complete functional testing, provides limited feedback while programming or cannot receive a replacement device. In such cases, knowing what degree electrode output is reduced can guide programming adjustments. However, appropriate clinical time must be arranged as procedures take 50–60 min per implant.

## 5 CONCLUSION

Electrodes with reduced output, as measured with surface potentials in‐vivo, show similar patterns when measured in‐vitro, hence validating that they are a measure of electrical output at each electrode. They are a useful additional test in the management of patients with suspected partial shorts. Once identified, patients who have received programming for suspected partial shorts should be monitored routinely for changes, with adjustments made to implant settings as required. At our centre, patients are monitored every 3–6 months depending on clinical concern. If a period of monitoring and reprogramming fails to improve CI functionality, re‐implantation is considered. The use of surface potentials in a CI audiologist's armamentarium aids in the clinical‐decision making process, particularly when considering re‐implantation.

## AUTHOR CONTRIBUTIONS

Susan T. Eitutis completed chart reviews, assisted with surface potential tests and wrote the paper. Yu Chuen Tam completed and analysed scalp surface potential measurements. Yu Chuen Tam, Iwan Roberts and Chloe Swords designed, performed and analysed *in‐vitro* surface potential validation checks. James R. Tysome, Neil P. Donnelly, Patrick R. Axon and Manohar L. Bance performed cochlear implant surgeries, reviewed X‐rays, and provided critical revision of the manuscript. The authors discussed the results and implications and commented on the work at all stages.

## FUNDING INFORMATION

The authors would like to thank Cambridge Hearing Trust for providing the equipment required to complete our testing. STE works for Cambridge University Hospitals as a clinical/research audiologist with funding provided by Advanced Bionics. CS is receiving funding from the Anatomical Society. JRT has previously received consultancy fees from Advanced Bionics and Oticon Medical. MLB is receiving funding from the MRC Confidence in Concept Fund and has previously received grant funding from MED‐EL, Advanced Bionics and Cochlear Corporation.

## CONFLICT OF INTEREST

The authors have no other conflicts of interest to disclose.

## Supporting information


**Supplementary Figure S1** Representative example of electrical dipole shifts affecting electrical output on surface potential measurements

**Supplementary Table S1** Clinical test outcomes, including electrode type and aetiology, for all patients showing a reduction electrical output

## Data Availability

Data that supports the findings of this study are available in the supplementary material of this article. Any additional data requests that support the findings of this study are available from the corresponding author upon reasonable request.
