# Supplementary material for: Detecting and managing partial shorts in Cochlear implants: A validation of scalp surface potential testing
Source: Clin Otolaryngol. 2022 Aug 1;47(6):641–9. doi: 10.1111/coa.13963 (PMC9804715; doi:10.1111/coa.13963)
Supplement: Supplementary file 1 — Supplementary Figure S1 Representative example of electrical dipole shifts affecting electrical output on surface potential measurements [file COA-47-641-s002.docx]

**Supplementary Figure 1:** Representative example of electrical dipole shifts affecting electrical output on surface potential measurements. A) Surface potential outputs when measured using ipsilateral (channel 1) and contralateral (channel 2) mastoids for electrodes 5, 14, 15 and 16. A decline in amplitude (blue) and reversal in output polarity (yellow) is present only when measured using the ipsilateral mastoid. B) Difference in electrical output across intra-cochlear electrodes when measured on ipsilateral (channel 1 – solid line) and contralateral (channel 2 – dotted line) mastoids. Measurements are shown for four control implants (i) and two implants with suspected partial short circuits (ii). Measurements for each electrode are presented as a percentage of the maximum electrical output measured for that implant ie.$(\frac{electrode output (\mu V)}{\max implant output (\mu V)})*100=normalised output (\%)$.
