# Supplementary material for: Detecting and managing partial shorts in Cochlear implants: A validation of scalp surface potential testing
Source: Clin Otolaryngol. 2022 Aug 1;47(6):641–9. doi: 10.1111/coa.13963 (PMC9804715; doi:10.1111/coa.13963)
Supplement: Supplementary file 2 — Supplementary Table S1 Clinical test outcomes, including electrode type and aetiology, for all patients showing a reduction electrical output [file COA-47-641-s001.docx]

**Supplementary Table 1:** Clinical test outcomes, including electrode type and aetiology, for all patients showing a reduction electrical output

| # | Implant type | Adult/paed | Aetiology | Communication mode | Impedance | NRI | SFAT | ASSE | BKB | Integrity test fault | Explant report |
| --- | --- | --- | --- | --- | --- | --- | --- | --- | --- | --- | --- |
| 1 | MS | paed | pre-lingual - unknown | oral | Drop e9-16 | not tested | Drop in high frequencies | Not tested | Not tested | Yes | confirmed V1 failure |
| 2 | MS | paed | pre-lingual - unknown | oral | Drop e12-16 | Loss of basal NRI | Drop in high frequencies | Not tested | Not tested | Yes | confirmed V1 failure |
| 3 | MS | paed | pre-lingual – unknown | oral | Drop 9-16 | Loss e9-16 | Drop in mid-high frequencies | Not measured | Not measured | Yes | confirmed V1 failure |
| 4 | MS | paed | pre-lingual - unknown | oral | Drop e13-16 | not tested | drop in high frequencies | No baseline | Not tested | Yes | confirmed V1 failure |
| 5 | MS | paed | pre-lingual - unknown | oral | Drop right e11-14 | not tested | stable* after increasing high frequency T/M levels | No baseline | Not tested | Yes | confirmed V1 failure |
| 6 | MS | adult | pre-lingual - measles | BSL | Drop in e9 -16 | not tested | Significant drop in high frequencies | Significant drop | BSL Not tested | NO (yes with more clinical evidence) | confirmed V1 failure |
| 7 | MS | paed | pre-lingual - connexin 26 | oral | Drop e13-15 | not tested | Drop in high frequencies | Drop | Drop L | Yes | confirmed V1 failure |
| 8 | MS | adult | post-lingual - unknown | oral | Drop 2018 e6-7 and 16 | not tested | Drop low and high frequencies | Drop | Drop | NO (continued progression) | report not yet returned |
| 9 | MS | adult | post-lingual -sudden hearing loss | oral | Drop in e12-14 | not tested | Drop | Drop | Drop | Yes | confirmed V1 failure |
| 10 | MS | adult | post-lingual - measles | oral | Drop e6, 8,10-16 | loss basal electrodes | Drop | Drop | Improved, limited data | Yes | confirmed V1 failure |
| 11 | SJ | adult | post-lingual - hereditary | oral | Drop e12-16 |  | Drop in high frequencies | Stable | Significant drop | NO (yes with more clinical evidence) | confirmed V1 failure |
| 12 | SJ | paed | pre-lingual - unknown | oral | Drop e9-16 electrodes | elevated/loss basal electrodes | Drop | No results | Too young | Yes | confirmed V1 failure |
| 13 | SJ | paed | pre-lingual - unknown | oral | Drop e13-16 electrodes | loss on basal electrodes | Drop | No results | Too young | Yes | confirmed V1 failure |
| 14 | SJ | adult | pre-lingual - unknown congenital | BSL + oral | Drop e9-16 electrodes | loss e9-16 | No baseline | No baseline | BSL user | Yes | confirmed V1 failure |
| 15 | SJ | adult | post-lingual - radiotherapy for neuroblastoma | oral | Drop e2,11, 13-16 | not tested | Drop | improved with programming | Not tested | 1st - no REPEAT - Yes | confirmed V1 failure |
| 16 | SJ | adult | post-lingual - unknown progressive | oral | Drop e12-16 | not tested | Stable | No baseline | Significant drop | Yes | confirmed V1 failure |
| 17 | MS | adult | post-lingual - unknown progressive | oral | Drop e9-16 | not tested | Drop high frequencies | Stable | Drop | Yes | confirmed V1 failure |
| 18 | MS | adult | pre-lingual - maternal rubella | oral + BSL | Drop e11-14, 16 | not tested | Drop high frequencies | Drop | Drop | Yes | Not explanted |
| 19 | MS | adult | post-lingual - hereditary | oral | Drop e1-7, 12-16 | not tested | Drop in low to mid frequencies | Stable | not tested | Yes | confirmed V1 failure |
| 20 | MS | adult | post-lingual - unknown progressive | oral | Drop 1-4, 13-16 | loss of NRI e5-16 | Drop in low and high frequencies | Drop 2019 | Limited data | Yes | Not explanted |
| 21 | MS | paed | pre-lingual - genetic? | oral | Drop e10-16 | loss of NRI e11-16 | Drop high frequencies | Not tested | Too young | Yes | confirmed V1 failure |
| 22 | MS | paed | pre-lingual - connexin 26 | oral | Drop e11-14, 16 | elevated/loss on basal electrodes | Drop high frequencies | Not tested | Too young | Yes | confirmed V1 failure |
| 23 | MS | paed | pre-lingual - connexin 26 | oral | Drop L e9-10, 14-16 | loss on basal electrodes | Drop L Sept 2019 ** no longer able to get NRIs | Not tested | Too young | Yes | confirmed V1 failure |
| 24 | MS | paed | pre-lingual - genetic? | oral | Drop e10-16 | not tested | Drop high frequencies | drop | not tested | Yes | confirmed V1 failure |
| 25 | MS | paed | pre-lingual - genetic? | oral | Drop e10 and 16 | loss of NRI e10-16 | stable | drop | not tested | NO (yes with more clinical evidence) | confirmed V1 failure |
| 26 | MS | paed | pre-lingual - unknown | oral | Drop e12-16 | loss e9-16 | Drop in high frequencies | Not tested | Too young | Yes | confirmed V1 failure |
| 27 | MS | paed | pre-lingual - unknown | oral | Drop e2-8 and 16 | loss of NRI across whole array | stable with programming, further drop Feb 2021 | limited information | drop Manchester picture test | NO (Yes 6 months later) | confirmed V1 failure |
| 28* | MS | paed | pre-lingual - unknown | oral | Open e2 and 12, with gradual drop 5-8, 10-11 not yet in fault criteria | loss of NRI e2, 9 | stable with programming | limited information | drop Manchester picture test | NO (continued progression) | report not yet returned |
| 29 | MS | adult | post-lingual - unknown progressive | oral | drop e9-16 | not tested | Drop high frequencies | stable | stable | Yes | confirmed V1 failure |
| 30 | SJ | paed | pre-lingual - suspected gentamycin | BSL | Drop e13-16 electrodes | loss e12-16 | Drop in high frequencies | not tested | not tested | Yes | confirmed V1 failure |
| 31 | MS | paed | pre-lingual - unknown | oral | Drop e13-16 electrodes | loss e16 | Drop in high frequencies | unable to do test | too young | Yes | confirmed V1failure |
| 32 | MS | paed | pre-lingual - unknown | oral | Drop e12-16 | elevated/loss e9-16 | Drop in high frequencies | unable to do test | too young | Yes | confirmed V1failure |
| 33 | SJ | paed | pre-lingual - unknown | oral | Drop e13-16 electrodes | elevated/loss e9-15 | unreliable | too young | Too young | Yes | confirmed V1failure |
| 34 | MS | adult | pre-lingual - unknown genetic | BSL | drop e4-5, 10-13 | not tested | not tested | reported drop in hearing, couldn’t' test in clinic | BSL | Yes | confirmed V1 failure |
| 35 | MS | adult | pre-lingual - CMV? | non-verbal, limited sign | Drop e12-16 | elevated/absent e12-16 | unreliable | unable to do test | non-verbal | Yes | confirmed V1 failure |
| 36 | MS | paed | pre-lingual - connexin 26 | oral | drop across whole array | elevated/loss all electrodes | Drop in low and high frequencies | not tested | Not measured | Yes | confirmed V1 failure |
| 37 | MS | paed | pre-lingual - genetic | oral | drop e10-16 | elevated/loss 9-16 | Drop low and high frequencies | not tested | Drop on AB words | Yes | confirmed V1 failure |
| 38 |  | paed | pre-lingual - genetic | oral | Drop e12-16 | loss of e12 | stable | not tested | drop in AB words at repeat visit | NO (evidence of further decline 6 months later) | Not explanted |
| 39 | SJ | adult | post-lingual - unknown | oral | Drop e9-16 | elevated/loss e7 and 10-16 | Drop in high frequencies | not tested | not measured | Yes | confirmed V1 failure |
| 40 | SJ | paed | pre-lingual - unknown | oral | Drop e13-14 | loss of NRI e13 | drop in high frequencies | too young | too young | Yes | confirmed V1 failure |
| 41 | SJ | paed | pre-lingual - 22q DiGeorge | oral | Drop e10-16 | elevated NRI e9-15 | Drop in high frequencies | not tested | too young | NO (yes with more clinical evidence) | confirmed V1 failure |
| 42 | MS | adult | pre-lingual - GJB2 | oral + BSL | Drop e14-16 electrodes | loss of NRI e9-16 | not tested | not tested | BSL | Yes | confirmed V1 failure |
| 43 | MS | adult | post-lingual - NIHL + otosclerosis | oral | Drop e1-6 | no responses | drop in low frequencies | drop | not tested | No (yes with more clinical evidence) | confirmed V1 failure |
| 44 | MS | adult | post-lingual - unknown | oral | Drop e9-16 | no responses | Drop in high frequencies | not repeated | drop | NO (yes 3 months later) | confirmed V1 failure |
| 45 | 3D | paed | pre-lingual - unknown | oral and sign | Drop e13-14 | loss of NRI e13-14 | not tested | too young | too young | NO (yes 6 months later with repeat EFI) | Not explanted |
| 46 | MS | adult | pre-lingual - CMV | non-verbal - Makaton | drop e13-16 and open e7 | elevated/loss e10-16 | unreliable | unable to test | unable to test | Yes | Not explanted |
| 47 | SJ | adult | post-lingual - familial + NIHL | oral | Drop e13-16 | not tested | Drop in high frequencies | not measured | Drop | Yes | confirmed V1 failure |
| 48 | MS | adult | post-lingual - presbycusis | oral | Drop e13-16 electrodes | elevated/loss e9-16 | Drop in high frequencies | Limited data | Limited data | Yes | confirmed V1 failure |
| 49 | MS | adult | pre-lingual - congenital hypoxia during birth | oral | Drop e12-16 | loss e9-16 | fluctuating | limited data | limited data | Yes | confirmed V1 failure |

Green highlights - implants where initial integrity testing was ‘normal’ but with further clinical evidence taken from surface potentials and functional information

Yellow highlights – implants were initial integrity testing was ‘normal’ and further clinical evidence was not accepted for device fault. However, progression resulted in the device being confirmed as faulty with Advanced Bionics integrity testing

Blue highlights – implants where initial integrity testing was ‘normal’ and further clinical evidence was not accepted for device fault. However, progression of changes are consistent with partial short circuits and are likely to be confirmed as a fault when follow-ups can be arranged

V1 Version 1 of the Advanced Bionics Ultra devices

* Suspected V1 failure with atypical presentation in impedances. Gradual decrease in impedances to nearly ½ but not quite meeting the criteria to be a ‘drop’, with additional 2 sudden ‘open’ circuits. Device was explanted with early signs of atypical device function without further monitoring as the patient was already undergoing surgery for a confirmed V1 failure on the contralateral ear.

MS Mid-Scala implant

SJ SlimJ implant

3D 3D SlimJ implant

NRI Neural responses

SFAT soundfield aided thresholds

ASSE Auditory speech sound evaluation

BKB Bamford-Kowal Bench sentences

EFI Electric field imaging
